# Outbreak of *Salmonella* Typhimurium linked to Swedish pre-washed rocket salad, Sweden, September to November 2022

**DOI:** 10.2807/1560-7917.ES.2024.29.10.2300299

**Published:** 2024-03-07

**Authors:** Karolina Fischerström, Rikard Dryselius, Mats Lindblad, Sharon Kühlmann-Berenzon, Nadja Karamehmedovic, Stefan Börjesson, Nasanin Hashemi, Ingrid Gunn, Ann-Mari Gustavsson, Nilla Lindroos, Joanna Nederby-Öhd, Micael Widerström, Ruska Rimhanen-Finne, Anni Vainio, Moa Rehn

**Affiliations:** 1 Public Health Agency of Sweden (PHAS), Solna, Sweden; 2 ECDC Fellowship Programme, Field Epidemiology path (EPIET), European Centre for Disease Prevention and Control (ECDC), Stockholm, Sweden; 3 Swedish Food Agency (SFA), Uppsala, Sweden; 4 School of Health Science, Örebro University, Örebro, Sweden; 5 Department of Infectious Disease Prevention and Control, Region Kalmar County, Kalmar, Sweden; 6 Department of Infection Prevention and Control, County of Värmland, Karlstad, Sweden; 7 Department of Infectious Disease Prevention and Control, Region Halland, Halmstad, Sweden; 8 Department of Infectious Disease Prevention and Control, Stockholm Region, Stockholm, Sweden; 9 Department of Clinical Microbiology, Umeå University, Umeå, Sweden; 10 Department of Health Security, Finnish Institute for Health and Welfare, Helsinki, Finland

**Keywords:** Salmonella, case-control study, outbreak, rocket salad

## Abstract

In September 2022, the Public Health Agency of Sweden observed an increase in domestic *Salmonella* Typhimurium cases through the Swedish electronic notification system, and an outbreak strain was identified with whole genome sequencing. Overall, 109 cases with symptom onset between 17 September and 24 November 2022 were reported from 20 of 21 Swedish regions. The median age of cases was 52 years (range 4–87 years) and 62% were female. A case–control study found cases to be associated with consumption of rocket salad (adjusted odds ratio (aOR) = 4.9; 95% confidence interval (CI): 2.4–10, p value < 0.001) and bagged mixed salad (aOR = 4.0; 95% CI: 1.9–8.1, p value < 0.001). Trace-back, supported by Finnish authorities who identified the Swedish outbreak strain in a Finnish cluster during the same time period, identified rocket salad, cultivated, pre-washed and pre-packed in Sweden as the likely source of the outbreak. No microbiological analyses of rocket salad were performed. Our investigation indicates that bagged leafy greens such as rocket salad, regardless of pre-washing procedures in the production chain, may contain *Salmonella* and cause outbreaks, posing a health risk to consumers. We emphasise the need for primary producers of leafy greens to identify possible contamination points to prevent outbreaks.

**Key public health message**

**What did you want to address in this study and why?**

*Salmonella* Typhimurium is one of the most common causes of food-borne gastrointestinal infections in Sweden, and in autumn 2022 an outbreak occurred. The investigation aimed to assess the extent of the outbreak, identify its source and initiate appropriate measures to prevent further cases and future outbreaks.

**What have we learnt from this study?**

This large *Salmonella* Typhimurium outbreak involved more than 100 confirmed cases in Sweden and another 20 cases in other Nordic countries. Swedish-produced rocket salad was the likely cause of the outbreak. Cross-border sharing of epidemiological, trace-back and microbiological data were used to identify the likely producer.

**What are the implications of your findings for public health?**

Regardless of pre-washing procedures in the production chain, bagged leafy greens may pose a health risk to consumers which emphasises the need to prevent contamination with *Salmonella* throughout the whole production chain.

## Background

In the European Union (EU), salmonellosis is the second most notified gastrointestinal infection among humans and a common cause of food-borne outbreaks [1]. In the years preceding the coronavirus disease 2019 (COVID-19) pandemic, salmonellosis notification rates in the EU were stable. In Sweden, salmonellosis is notifiable by law [2]. In 2015–2019, the average number of annually notified cases in Sweden was 2,171 (range 1,992–2,297) with about two thirds being reported as infected abroad [3]. The most common serovars related to outbreaks are *Salmonella* Enteritidis and Typhimurium including its monophasic variant.

Food-borne outbreaks of salmonellosis are typically linked to eggs, chicken meat and pork products [4,5]. Additionally, food-borne outbreak data in several countries suggest that raw vegetables, including leafy greens, are an important source and the European Food Safety Authority (EFSA) has ranked *Salmonella* and leafy greens as the highest priority among specific food/pathogen combinations linked to food-borne infections from food of non-animal origin [6]. Risk factors for contamination of leafy greens with *Salmonella* in primary production include environmental factors, such as heavy rainfalls causing floods, animals (domestic or wildlife), use of untreated manure, contaminated agricultural water, and cross-contamination by food handlers and equipment at harvest or on farm post-harvest [7].

## Outbreak detection

At the end of September 2022, the Public Health Agency of Sweden (PHAS) observed an increase of domestic *Salmonella* cases reported by treating physicians and clinical microbiological laboratories through the Swedish national electronic notification system for notifiable diseases (SmiNet). Data obtained from regional clinical microbiological laboratories performing subtyping of *Salmonella* isolates on serogroup level suggested that *Salmonella* serogroup B was causing the national increase of cases.

On 4 October 2022, PHAS identified a cluster of eight cases of *S.* Typhimurium belonging to the 7-gene multilocus sequence type (ST) 19 based on whole genome sequencing (WGS), suggesting a common source. The cluster was identified as part of the PHAS microbial surveillance programme, which is based on voluntary submission of metadata and isolates of *Salmonella* from domestic cases, by the regional clinical microbiological laboratories. At PHAS, typing is performed using WGS and conventional slide agglutination can be done if needed.

On 7 October 2022, a national outbreak was declared, following the laboratory confirmation of another 11 cases belonging to the cluster distributed in 11 of 21 Swedish regions. The outbreak control team was coordinated by PHAS and included the Swedish Food Agency (SFA) and regional departments of communicable disease control and prevention (CDC).

We describe an outbreak investigation of *S.* Typhimurium in Sweden linked to cases in Finland, in September–November 2022. We collaborated with Finnish authorities and aimed to assess the extent of the outbreak, identify its source and initiate appropriate control measures to prevent further cases.

## Methods

### Outbreak case definition and case finding

We defined a confirmed case as a person with laboratory-confirmed *S*. Typhimurium, clustering with the outbreak strain within five single nucleotide polymorphisms (SNPs) based on WGS, notified from 10 September 2022 and onwards and with no recent travel history.

We defined a probable case as a person with laboratory-confirmed *Salmonella* serogroup B infection, notified from 10 September 2022 and onwards and with no recent travel history.

Cases were reported to SmiNet through passive routine surveillance with mandatory notifications by both laboratories and clinicians. The reported case data included age, sex, region, date of symptom onset, date of sampling and suspected country of infection. If date of symptom onset was missing, date of sampling was used instead.

To speed up the investigation and identification of probable cases, on 10 October, PHAS informed all regional clinical microbiological laboratories in Sweden about the ongoing *S.* Typhimurium outbreak and requested the laboratories performing serogrouping of *Salmonella* isolates to report this result as metadata linked to the isolate before sending them to PHAS. Additionally, PHAS performed serogroup identification using conventional slide agglutination before WGS on isolates received from laboratories that did not perform serogrouping of *Salmonella*.

### Epidemiological investigation

#### Trawling questionnaire and population survey on eating habits

To gather information on food and environmental exposures, we used a web-based standardised trawling questionnaire routinely used for investigation of *Salmonella* cases, consisting of inquiries on 76 exposures during the 7 days before symptom onset. The trawling questionnaire was completed by cases online or by regional CDCs via phone interviews with cases. In 2010–2011, the SFA conducted a survey on eating habits in Sweden [8]. To identify exposures that were statistically more common among our cases than in the Swedish population, for each item in the trawling questionnaire, we calculated the probability with 95% confidence intervals (CI) of the observed number of cases exposed to the item, given the expected proportion of exposed in the population from the SFA survey [9].

We selected exposures to investigate further in a case–control study based on the following: (i) the exposure was significantly more common among cases than in the Swedish population according to the SFA survey on eating habits (see above) and > 10% of cases were exposed according to the trawling questionnaire or (ii) > 50% of cases were exposed according to the trawling questionnaire, regardless of exposure prevalence in the population.

#### Case–control study

We performed a case–control study to test the hypothesis that one of the selected food exposures was the source of infection. All confirmed and probable cases were eligible for inclusion. We aimed to select 10 controls per case from a national random pool of controls available at PHAS, matched on sex and age group (2–5, 6–12, 13–18, 19–29, 30–49, 50–69 and ≥ 70 years).

#### Data collection

We created a web-based questionnaire on exposures during the 7 days preceding symptom onset for cases and the 7 days preceding the response for controls. We included the following exposures based on the selection criteria described above: the grocery retailers, apples, bagged mixed salad, carrots, cashew nuts, cucumber, fresh herbs, medium tomatoes, onion, pepper, pumpkin seeds, rocket salad, small tomatoes, sugar snaps and walnuts. Respondents could answer ’Yes‘, ’Probably‘, ’Probably not‘ or ’No‘. We also included open-ended questions to receive detailed information on the consumed products. For cases, we included questions on hospitalisation and presence of the following symptoms: fever, stomach pain, diarrhoea, bloody diarrhoea or vomiting.

The web-based questionnaire was completed by cases online or via phone interviews conducted by regional CDCs and by controls online.

#### Data analysis

We included only confirmed cases in the case–control analysis. We described the distribution of cases by region, age, sex and date of symptom onset for all confirmed cases and symptoms and hospitalisation among cases in the case–control study.

As many matched groups lacked responses from the case or controls, decreasing the statistical power, we performed an age and sex adjusted unmatched analysis with all cases and controls that had responded to the questionnaire.

For analysis of exposures, the answer ’Probably‘ was re-categorised as ’Yes‘ and ’Probably not‘ was re-categorised as ’No‘. We calculated adjusted odds ratios (aOR) for age and sex with 95% CIs using exposure-specific univariable logistic regression models. Exposures with > 30% of cases exposed, odds ratios (OR) > 1 and p value < 0.2 were included in a multivariable logistic regression along with age and sex. In the complete-case multivariable analysis, p value < 0.05 was considered statistically significant. We applied stepwise backward selection, where non-significant exposures were removed according to a likelihood ratio test. The final model included statistically significant exposures, age and sex. In addition, we examined interactions between food exposures in the final model. Data analyses were performed with the Stata 16.1 software (StataCorp LLC, United States (US)).

#### Receipts of food purchase

Confirmed cases were asked to send grocery receipts from the week preceding symptom onset, regardless of whether they responded to the questionnaire or not. Information on the date of purchase and the grocery retailer identified in the case–control study were documented and used in the trace-back investigation.

### Microbiological investigation and antimicrobial resistance profile

Clinical specimens were routinely analysed by regional microbiological laboratories and isolates of *Salmonella* species were sent to PHAS for further typing. When serogrouping of *Salmonella* was conducted at PHAS, conventional slide agglutination according to the White-Kauffmann-Le Minor scheme was performed [10]. The DNA was extracted using the PSS magLEAD 12gC (Precision System Science Co., Ltd, Matsudo, Japan) after which library preparation was performed using Ion Xpress Plus Fragment Library Kit for AB Library Builder System (Thermo Fisher Scientific, Waltham, US). Sequencing was performed on an Ion S5 XL System and an Ion GeneStudio S5 Prime System (Thermo Fisher Scientific).

To define species, the Basic Local Alignment Search Tool (BLAST) against local databases with reference genomes was used and ST was determined by the Achtman 7-gene multilocus sequence typing (MLST) scheme [11-13]. Serovar prediction was done using SeqSero and an in-house database of STs linked to serovars [14]. All outbreak isolates were tested for antimicrobial resistance (AMR) markers using AMRFinderPlus v3.11.18 [15]. The SNP analysis was calculated by MSTgold and visualised by minimal spanning trees [16].

To enable sharing of sequence data internationally via Enterobase (https://enterobase.warwick.ac.uk) and EpiPulse, the European Centre for Disease Prevention and Control (ECDC) online portal for European surveillance of infectious diseases, DNA from an isolate representative of the outbreak was submitted to Clinical Genomics, Science for Life Laboratory, Solna, Sweden, for sequencing using Illumina NovaSeq 6000 (Illumina Inc., San Diego, US).

The regional CDCs asked cases for leftovers of suspected food samples to be sent for microbiological analysis.

#### International investigation

On 18 October, PHAS uploaded the Illumina-generated raw reads of the representative isolate to Enterobase (SAL_OB9498AA) and posted an event (2022-FWD-00082) in EpiPulse to inquire if other countries had observed cases with the outbreak strain. Countries that reported cases with isolates closely related to the outbreak strain were contacted by PHAS for further details.

### Trace-back investigation

The SFA conducted a trace-back investigation of the implicated products based on the results of the case–control study, food purchases and data from a simultaneous outbreak in Finland with the Swedish outbreak strain.

## Results

### Epidemiological investigation

#### Descriptive epidemiology

**Figure 1:**
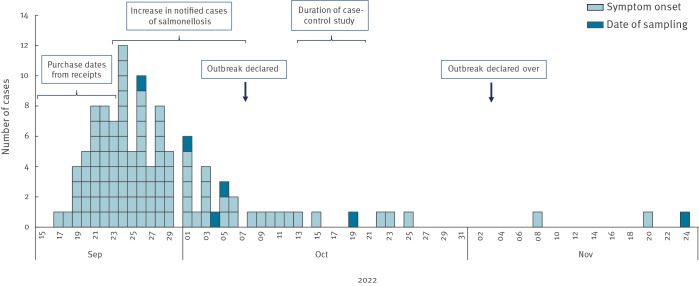
Confirmed cases of *Salmonella* Typhimurium sequence type 19 in an outbreak linked to pre-washed rocket salad, by date of symptom onset or date of sampling, Sweden, September–November 2022 (n = 109)

**Figure 2:**
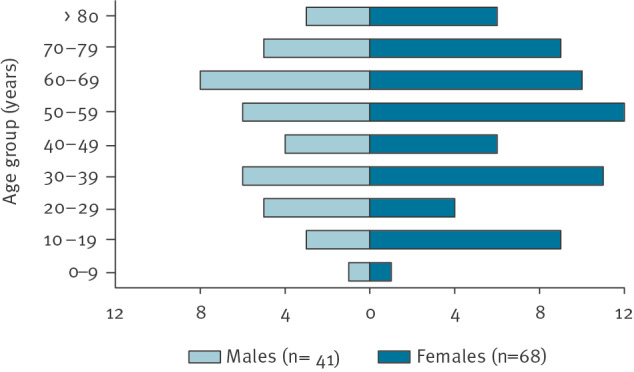
Confirmed cases of *Salmonella* Typhimurium sequence type 19 in an outbreak linked to pre-washed rocket salad, by age group and sex, Sweden, September–November 2022 (n = 109)

In total, 109 confirmed cases reported symptom onset between 17 September and 24 November 2022 (Figure 1). The median age of cases was 52 years (range 4–87 years) and 62% were female (Figure 2). Cases were reported from 20 of the 21 Swedish regions. Two regions reported more than 10 cases each. Frequently reported symptoms among 52 cases responding to the questionnaire were diarrhoea (n = 51), fever (n = 49), stomach pain (n = 46), bloody diarrhoea (n = 18) and vomiting (n = 15), and 22 of these cases were hospitalised.

### Case–control study

Between 14 and 21 October 2022, 54 cases (50%) and 434 controls (37%) responded to the questionnaire and were included in the analysis.

In the univariable analysis, rocket salad and bagged mixed salad had the highest ORs and were included in the multivariable analysis with additional six other exposures (Table 1). The final multivariable model included rocket salad, bagged mixed salad and shopping at Grocery retailer A. An additional model including an interaction between rocket salad and bagged mixed salad suggested a stronger association between exposure and illness when both rocket salad and bagged mixed salad were consumed, compared with when only rocket salad, only bagged mixed salad, or neither of the two were consumed (Table 2).

**Table 1:** Number and percentage of exposed cases and controls and adjusted odds ratios in univariable and multivariable models in an investigation of an outbreak of *Salmonella* Typhimurium sequence type 19 linked to pre-washed rocket salad, Sweden, September–November 2022

Exposure	Cases (n = 54)		Controls (n = 434)		Univariable model			Final multivariable model				
	n	%^a^	n	%^a^	aOR^b^	95% CI	p value	aOR^c^	95% CI			p value
Food items												
Apples	21	42	169	39	1.1	0.59–2.1	0.703	Not included				
Bagged mixed salad^d^	30	63	90	21	6.3	3.2–12	< 0.001	4.0	1.9–8.1			< 0.001
Carrots	30	60	224	52	1.4	0.72–2.6	0.312	Not included				
Cashew nuts	8	16	72	17	0.94	0.37–2.2	0.888					
Cucumber^d^	37	73	264	62	1.7	0.84–3.4	0.124					
Fresh herbs	12	24	73	17	1.6	0.70–3.2	0.215					
Medium tomatoes^d^	25	48	160	37	1.6	0.85–2.9	0.119					
Onion	34	69	305	71	0.92	0.47–1.9	0.803					
Pepper^d^	32	64	204	47	2.0	1.0–3.8	0.027					
Pumpkin seeds^d^	16	33	81	19	2.1	1.0–4.1	0.023					
Rocket salad^d^	30	60	71	17	7.6	3.9–15	< 0.001	4.9		2.4–10	< 0.001	
Small tomatoes^d^	37	71	251	58	1.8	0.92–3.6	0.070	Not included				
Sugar snaps	8	16	17	4	4.8	1.7–12	< 0.001					
Walnuts	8	16	57	13	1.3	0.49–2.9	0.552					
Grocery retailer												
Grocery retailer A^d^	48	89	327	75	2.6	1.1–7.7	0.026	3.1		1.0–9.5	0.043	
Grocery retailer B	17	31	176	41	0.67	0.34–1.3	0.199	Not included				
Grocery retailer C	7	13	118	27	0.40	0.15–0.92	0.024					
Other grocery retailers^e^	6	11	16	4	3.3	0.99–9.3	0.013					

aOR: adjusted odds ratio; CI: confidence interval.

^a^ Percentage exposed of those who answered the question.

^b^ Adjusted for age and sex.

^c^ Stepwise backward selection, adjusted for age and sex.

^d^ Included in the multivariable analysis.

^e^ 18 grocery retailers in the questionnaire grouped as one variable.

**Table 2:** Number and percentage of exposed cases and controls, and adjusted odds ratios in multivariable model with interactions in an investigation of an outbreak of *Salmonella* Typhimurium sequence type 19 linked to pre-washed rocket salad, Sweden, September–November 2022

Exposure	Cases (n = 54)		Controls (n = 434)		Final model with interaction		
	n	%^a^	n	%^a^	aOR^b^	95% CI	p value
Rocket salad and mixed salad bags	19	40	37	9	22	8.6–55	< 0.001
Rocket salad, no mixed salad bags	10	21	34	8	13	4.6–36	< 0.001
Mixed salad bags, no rocket salad	11	23	52	12	9.5	3.5–25	< 0.001
No rocket nor mixed salad bags	8	17	303	71	Reference		
Grocery retailer A	48	89	327	75	3.1	1.0–9	0.045

aOR: adjusted odds ratio; CI: confidence interval.

^a^ Percentage exposed of those who answered the question.

^b^ Adjusted for age and sex and the other variables in the model.

#### Receipts of food purchase

We received 69 receipts from 21 confirmed cases. The receipts were dated between 12 September and 2 October. Only receipts from Grocery retailer A (n = 65) were included in the investigation. Seventeen cases had receipts that included bagged rocket salad (n = 8) and/or mixed salad (n = 11). These purchases took place on 15–17 and 16–23 September, respectively.

### Microbiological investigation and antimicrobial resistance profile

**Figure 3:**
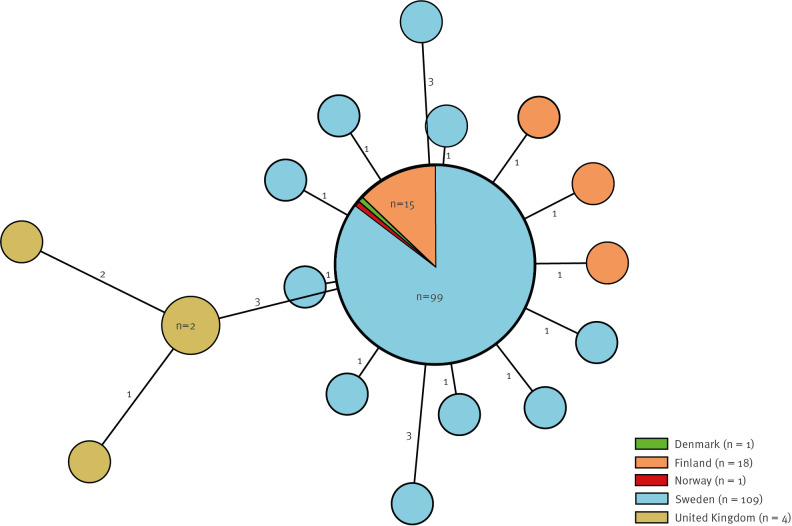
Minimum spanning tree of *Salmonella* Typhimurium sequence type 19 isolates from Denmark, Finland, Norway, Sweden and the United Kingdom, August 2021–January 2023 (n = 133)

Of the 109 isolates from confirmed Swedish cases, 107 (98%) clustered within 0–2 SNP differences (Figure 3). The outbreak strain was not closely related (≥ 170 SNPs) to any other isolate in the PHAS WGS database consisting of isolates routinely sequenced within the microbial surveillance programme since April 2019. None of the isolates carried transferable genes encoding AMR.

Numbers of single nucleotide polymorphisms (SNPs) are shown next to the branches (not proportional in length to differences in SNPs). Colours demonstrate the country of origin of the isolate.

No food samples of suspected products from cases were available for laboratory analysis. As the outbreak was over by the time the trace-back investigation identified the producer, no attempts were done to sample products at the producer level.

#### International investigation

Of the 11 countries responding to the event in EpiPulse, four reported cases: Finland (n = 18), the United Kingdom (UK) (n = 7), Denmark (n = 1) and Norway (n = 1). The requested WGS raw reads from the responding countries were closely related to the outbreak strain: Finland 0–1 SNPs, Denmark and Norway no SNP differences and the UK 3–5 SNPs (Figure 3). No genes encoding AMR were detected in the isolates from Denmark, Finland and Norway, while the isolates from the UK were positive for *bla*_TEM-1_, *sul2*, *dfrA5*, *aph*(*3”)-Ib* and *aph(6)-Id*. The isolates from the Nordic countries and the UK had the plasmid replicons IncFIB, IncFII and IncX1, while all the UK isolates also contained a partial IncQ1.

The 18 cases in Finland were reported from five Finnish hospital districts: 17 in September–October 2022 and one in January 2023. Two cases were part of a local cluster in which six persons became ill after dining at a restaurant in Western Finland. The Finnish authorities shared their findings directly with the Swedish outbreak team.

The Norwegian case had a sampling date of 28 September 2022 and had recently travelled to Sweden. The Danish case had a sampling date in late September 2022 with no additional information. The UK cases had sampling dates between July 2021 and February 2022 and no travel to the Nordic countries was reported.

#### Trace-back investigation

The trace-back investigations showed that bagged rocket salad and mixed salad including rocket salad sold at the Grocery retailer A stores were all delivered from one packaging company in Sweden. In September 2022, this company exclusively bought rocket salad from three growers within the same region in southern Sweden. These three growers delivered rocket salad in total quantities of 16–21 tons per week to the packaging company. At the packaging company, all leafy greens went through a washing process with potable water consisting of two washing steps and a final rinse before the salad was dried and packaged. There were no separate washing tanks for rocket salad. Instead, all leafy greens were washed in the same tanks.

**Figure 4:**
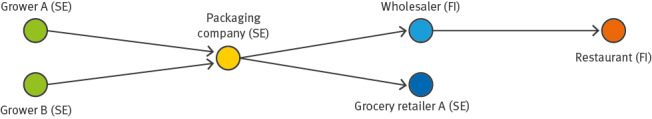
Schematic overview of the production chain of rocket salad in the trace-back investigation of an outbreak of *Salmonella* Typhimurium sequence type 19 linked to pre-washed rocket salad, Sweden and Finland, September–November 2022

FI: Finland; SE: Sweden.

The Finnish food control authorities identified a particular batch of rocket salad as the likely source of the Finnish outbreak. The rocket salad originated from the same packaging company in Sweden, previously identified in the Swedish trace-back investigation. This batch included rocket salad from two of the three Swedish growers delivering rocket salad to the packaging company (Figure 4).

The figure was created with FoodChain-Lab (https://foodrisklabs.bfr.bund.de/foodchain-lab/), an open-source software developed by the German Federal Institute for Risk Assessment (BfR).

## Outbreak response measures

On 10 October, PHAS published information about the outbreak on their website. The information was regularly updated during the outbreak to sustain awareness of the ongoing outbreak until the outbreak was declared over on 3 November.

Once the preliminary results of the case–control study were available, they were forwarded to Grocery retailer A for further investigation on the implicated product. Grocery retailer A received information from the packaging company that *Salmonella* had not been found in the samples from their routine analysis of the produce.

The outbreak had already stopped when the suspected source of the outbreak was identified. Possible contaminated batches of bagged rocket or mixed salad were not likely to be on the market anymore.

The two growers identified in the trace-back investigation were contacted by the regional County Administrative Board to investigate possible sources of *Salmonella* contamination. Both growers exclusively used mineral fertilisers and there were no pronounced problems with wildlife or flooding during the growing season. One grower used irrigation water from ponds and streams and applied ultraviolet (UV) radiation for disinfection. The other grower used irrigation water from deep drilled wells. The latest water samples taken by the growers were analysed in August and May 2022, respectively, without any indication of contamination. In addition, there were no findings of *Salmonella* in the most recent monthly routine samplings of rocket salad from the packaging company.

## Discussion

We describe, to our knowledge, one of the largest national *Salmonella* outbreaks in Sweden linked to a Swedish vegetable produce. The outbreak occurred during the autumn of 2022 and involved 109 confirmed cases spread throughout Sweden and additional 20 cases in other Nordic countries. The quickly transient peak of the epidemic curve indicated that a fresh product with a short shelf-life was the vehicle of the outbreak. A vegetable source was suggested based on the trawling questionnaires and the dominance of female cases as well as the overall age distribution [17]. Although not confirmed microbiologically, epidemiological evidence and findings from a thorough trace-back investigation supported by the Finnish authorities, suggested Swedish cultivated rocket salad as the common source. We identified one Swedish packaging company of rocket salad that also included rocket salad in their mixed salad bags, and that delivered bagged rocket and mixed salad to the suspected grocery retailer during the outbreak period. Self-reported consumption of rocket salad, consumed alone or in mixed salad bags, could explain 83% of the cases.

Our conclusions were supported by findings from the Finnish outbreak investigation, independent of the Swedish one, which identified rocket salad from the same packaging company in Sweden as the likely source. Through successful collaboration with the Finnish investigators, the possible source of contaminated rocket salad could be narrowed down to two growers of rocket salad in southern Sweden.

Apart from Finland, there were also cases with related isolates notified in Norway, Denmark and the UK. Besides the report of the Norwegian case visiting Sweden, no additional epidemiological information was available. Due to the proximity between Denmark and southern Sweden, travel to Sweden and/or consumption of Swedish fresh produce is not unlikely. The UK cases, however, were not likely to be linked to this outbreak as the cases were notified 7–14 months before the Swedish and Finnish outbreaks and no travel to the Nordic countries was reported. The UK isolates also differed by carrying AMR genes. The possible epidemiological link between the UK and the Nordic isolates remains unknown. Although not indicated in our investigation, we speculate that an environmental reservoir for the outbreak strain exists, for example surface waters or wildlife, such as gulls. Gulls in Sweden have been reported to be carriers of *S*. Typhimurium isolates similar to those found in humans, and they have been shown to migrate between the UK and Sweden [18,19].

Risks for transmission of *Salmonella* from non-animal origin are already well-described, and bagged salad and rocket salad have previously been associated with outbreaks of other pathogens, such as Shiga toxin-producing *Escherichia coli*, *Yersinia enterocolitica* and *Cryptosporidium parvum* [6,20-26]. Pre-washed and bagged salads pose a risk of infection despite thorough cleaning procedures by packaging companies [22]. Pre-washing procedures at the packaging company in this outbreak did not eliminate *Salmonella* from the rocket salad delivered to Grocery retailer A. However, contamination of the rocket salad most likely did not occur during pre-washing, because other leafy greens were washed in the same tanks and there were no signs of any other leafy green separately causing infection except for rocket salad. More likely, contamination occurred in one or several fields of the two implicated growers. The use of fertilisers, flooding or wildlife were not considered as likely sources of contamination, and routine water samples taken earlier in the growing season, both from deep drilled wells and from surface water after UV purification, did not indicate any problems. However, in a previous survey on Swedish irrigation water, faecal indicator organisms were identified after UV purification of surface water from rivers and ponds [27], suggesting that the irrigation water still may have been contaminated. Also, the possibility remains that crop was contaminated by wild animals, such as seagulls and deer, even though no major populations of wild animals in the vicinity of the fields had been observed.

*Salmonella* can persist and proliferate in various types of ready-to-eat vegetables, including rocket salad, emphasising the importance of maintaining temperatures < 7°C during commercialisation [28]. The moist environment in bagged salads due to juices released from chopped salad leaves facilitates colonisation of *Salmonella* and enhances pathogen attachment to the content of the bag, even in refrigerated conditions [29]. Once *Salmonella* cells have contaminated leafy greens, they are difficult to get rid of by washing [30]. Temperature control is therefore crucial to minimise pathogen growth [31,32]. In this investigation, we unfortunately did not have any information about storage temperatures at Grocery retailer A. Improperly stored bags could lead to increased levels of *Salmonella* and to reduce the risk of food-borne illness and prevent food waste, SFA recommends that consumers should keep the refrigerator temperature at 4°C [33], but leafy greens may still be stored at higher temperatures than recommended [34]. Future outbreak investigations may consider including questions on consumption dates and how bags were stored to better understand how storage conditions affect the risk of infection.

This investigation had the following limitations. We were not able to obtain any microbiological evidence from food samples, neither from leftovers from the cases nor from Grocery retailer A or at the producer level. This is not uncommon when epidemiological evidence points to leafy greens as the suspected source of an outbreak, since leafy greens are often consumed quickly or discarded, limiting opportunities for sampling and analysis in a timely manner [35]. Neither did we obtain samples from the irrigation water at the implicated growers. Another limitation was recall bias that could have occurred among both cases and controls, although cases were assumingly more likely to remember what they had eaten the days before becoming ill compared with controls that did not have a specific event to relate their food consumption to. Finally, we did not perform a matched analysis, although we did a matched design in selection of cases and controls. The reason for this was non-response among both cases and controls. Five cases were matched to non-responding controls, and 228 controls were matched to non-responding cases. Instead, in our unmatched analysis, we adjusted for the matching variables (age and sex) to account for bias. Additionally, as sensitivity analysis, we performed a matched analysis using conditional logistic regression on the subset of matched data and obtained similar results (data not shown).

## Conclusion

Our investigation suggests that rocket salad produced in Sweden was the source of this large *S.* Typhimurium outbreak. Multidisciplinary and international collaboration between national and regional public health and food safety authorities found evidence to support the conclusion but no microbiological confirmation was obtained. This investigation therefore highlights the public health value of multi-country sharing of epidemiological, trace-back and microbiological data. Leafy greens are likely to be consumed raw, which presents challenges in ensuring microbial safety throughout the whole production chain. Our investigation indicates that, regardless of pre-washing procedures in the production chain, bagged leafy greens such as rocket salad, may contain *Salmonella* and cause outbreaks, posing a health risk to consumers. We emphasise the need for primary producers of leafy greens to identify possible contamination points to prevent outbreaks.
